# Overcoming challenges of implementation of FBT at the Regional Eating Disorders Service (REDS) in Auckland, New Zealand

**DOI:** 10.1186/2050-2974-2-S1-O16

**Published:** 2014-11-24

**Authors:** Roger Mysliwiec, Maugan Rimmer

**Affiliations:** 1 Regional Eating Disorders Service, Auckland, New Zealand

## 

In 2010 the New Zealand Ministry of Health made a decision to roll out training in FBT across New Zealand for clinicians working with eating disorders. The expectation was that the three regional specialist services in New Zealand would subsequently provide a lead to ensure implementation of FBT across their Region.

This presentation will describe the process of change to achieve uptake of FBT as a new treatment model by the existing adolescent team of the Regional Eating Disorder Service in Auckland (REDS). The steps taken to overcome the significant initial challenges and the strategies and the resources required to achieve a sustainable model of provision of FBT at REDS will be discussed as well as its implications for regional training and supervision of CAMHS clinicians across the Northern and Midland Regions of New Zealand.

The positive impact of these changes on outcome data is described in more detail in the presentation "What difference does FBT make? Results of a service audit following the implementation of FBT".

This abstract was presented in the **Service Initiatives: Child and Adolescent Refeeding and FBT** stream of the 2014 ANZAED Conference.

